# Systematic Review of Primary Immunodeficiency Diseases in Malaysia: 1979–2020

**DOI:** 10.3389/fimmu.2020.01923

**Published:** 2020-08-26

**Authors:** Intan Juliana Abd Hamid, Nur Adila Azman, Andrew R. Gennery, Ernest Mangantig, Ilie Fadzilah Hashim, Zarina Thasneem Zainudeen

**Affiliations:** ^1^Primary Immunodeficiency Diseases Group, Regenerative Medicine Cluster, Institut Perubatan and Pergigian Termaju, Universiti Sains Malaysia, Kepala Batas, Malaysia; ^2^Department of Biomedical Science, Universiti Islam Antarabangsa, Kuantan, Pahang, Malaysia; ^3^Sir James Spence Professor of Child Health, Translational and Clinical Research Institute, Newcastle University, Great North Children's Hospital, Newcastle upon Tyne, United Kingdom

**Keywords:** primary immunodeficiency, inborn error of immunity, epidemiology, prevalence, Malaysia

## Abstract

**Introduction:** Primary immunodeficiency diseases (PIDs) are under-reported in Malaysia. The actual disease frequency of PID in this country is unknown due to the absence of a national patient registry for PID.

**Objective:** This systematic review aimed to determine the prevalence rates of PID cases diagnosed and published in Malaysia from 1st of January 1979 until 1st of March 2020. It also aimed to describe the various types of PIDs reported in Malaysia.

**Method:** Following the development of a comprehensive search strategy, all published literature of PID cases from Malaysia was identified and collated. All cases that fulfilled the International Union of Immunological Societies (IUIS) classification diagnosis were included in the systematic review. Data were retrieved and collated into a proforma.

**Results:** A total of 4,838 articles were identified and screened, with 34 publications and 119 patients fulfilling the criteria and being included in the systematic review. The prevalence rate was 0.37 per 100,000 population. In accordance with the IUIS, the distribution of diagnostic classifications was immunodeficiencies affecting cellular and humoral immunities (36 patients, 30.3%), combined immunodeficiencies with associated or syndromic features (21 patients, 17.6%), predominant antibody deficiencies (24 patients, 20.2%), diseases of immune dysregulation (13 patients, 10.9%), congenital defects in phagocyte number or function (20 patients, 16.8%), defects in intrinsic and innate immunity (4 patients, 3.4%), and autoinflammatory disorders (1 patient, 0.8%). Parental consanguinity was 2.5%. Thirteen different gene mutations were available in 21.8% of the cases.

**Conclusion:** PIDs are underdiagnosed and under-reported in Malaysia. Developing PID healthcare and a national patient registry is much needed to enhance the outcome of PID patient care.

## Introduction

Primary immunodeficiency diseases (PIDs) or inborn errors of immunity are inherited disorders that impair the immune response, leading to increased risk of infections, immune dysregulation, autoimmune phenomena, inflammation, and malignancy. They are classified as “rare diseases,” but their global incidence is more prevalent than generally thought (1). Six million people are estimated to be living with PIDs worldwide, of which only 27,000–60,000 have been diagnosed (2). Early detection and prompt action by clinicians could reduce complications and save lives by administering appropriate treatment.

Malaysia is a small country situated in Southeast Asia, with a total population of 32.6 million; the total livebirths in 2018 was 501.9 thousand, and the GDP per capita is 10,940 USD. The estimated life expectancy in Malaysia is 74.5 years, and 28% of the population is aged less than 18 years. The under-5 mortality rate was 7.8 deaths per 1,000 live births in 2018 (3).

Management and research of PIDs in Malaysia are met with many challenges. Vast gaps in knowledge of PIDs and translation of diagnosis and management into clinical practice exist. Given the scarce epidemiological PID data and the lack of patient awareness or diagnostic facilities in Malaysia, PIDs are a tremendous challenge for the biomedical community, thereby causing unnecessary suffering, and deaths. Therefore, national studies must be considered to efficiently and systematically assess the proportion of affected individuals amongst the general population (4).

Furthermore, estimates of PIDs frequency in Malaysia are needed to determine the cost-effectiveness of routine screening. Most incidence and prevalence determination is performed using data from national patient registry databases (5, 6). Given the absence of a Malaysian PID registry database, the prevalence rate was calculated in the present paper by systematically reviewing PID literature from Malaysia to provide retrospective data for the medical community, the health authorities, and PID patients and their families and improve the knowledge of PIDs. This systematic review offers a mechanism to describe the epidemiological landscape of PIDs in Malaysia and constitutes an opportunity to determine the prevalence rates of PIDs in a defined non-referred population.

## Methods

**Figure 1:**
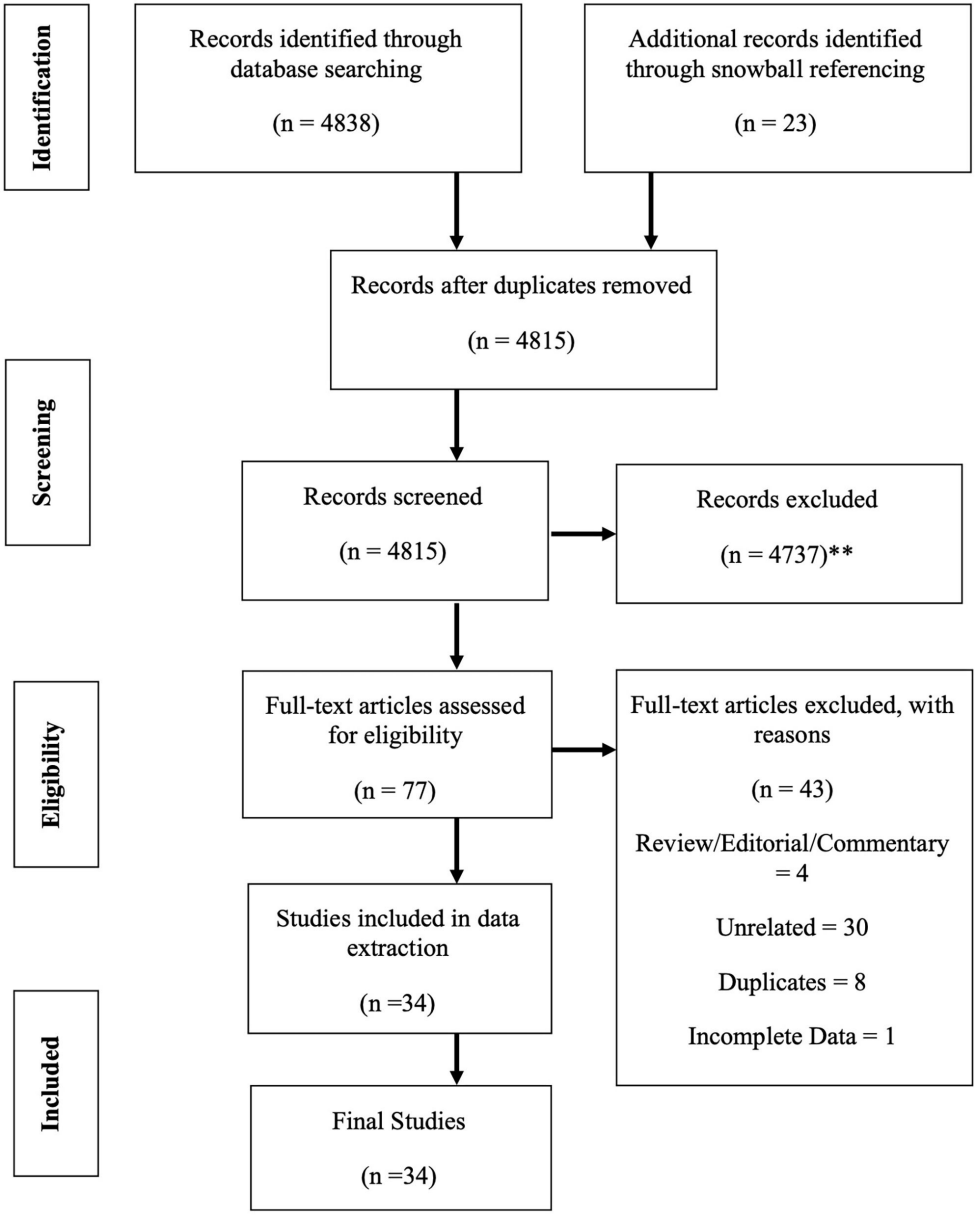
PRISMA flow diagram: the PRISMA (Preferred Reporting Items for Systematic Review and Meta-Analysis) diagram details our search and selection process applied during the overview. (**Reasons excluded are listed in Supplement 1).

In these sections, the strategy to identify relevant citations gathered for this review is described, and a summary of the results is provided. A comprehensive search of the Cochrane Library and Medline databases (via PubMed) was initially conducted from its inception to 1st of March 2020. A title/abstract keyword search was conducted, 77 candidate article abstracts were manually reviewed, and forward/backward reference searches were completed. Controlled vocabulary supplemented with keywords (which included autoimmunity) was used to search for individual primary immunodeficiencies in accordance with the IUIS 2019 classification (7). The search results and study selections are outlined in Figure 1. The PICO statement was as follows: P, patients with PID in Malaysia; I, none considering looking into the epidemiology and prevalence study; C, other neighboring Southeast Asian countries and O, prevalence and incidence rates.

Cases were screened using primary immunodeficiencies in accordance with the IUIS 2019 classification (7). For all published papers retrieved, the second, fifth, and sixth author reviewed the title and abstracts independently and performed the selections on the basis of pre-specified inclusion and exclusion criteria. All inclusions and exclusions were discussed and finalized with all team members. The primary author reviewed the clinical profile, laboratory parameters, other comorbid conditions, and other associated factors discussed in the papers and retrieved the relevant information. Age and year at diagnosis were retrieved from the publications. In cases where year was not listed, an assumption that the year of diagnosis was equal to the year of publication was made in specific articles/cases. Attempts were also made to contact the original authors of the included published studies via email in cases of incomplete information or for further clarification. The prevalence rates were calculated by the fourth author. The third author offer critical review on the manuscript draft.

The agreement of reviewers on the methodological quality assessment was assessed, and any disagreement was resolved by discussion or, if agreement could not be reached, by arbitration by the third author. Reviewers were not masked to study details when assessing the study quality.

The period prevalence rate per 100,000 population was calculated as the number of all existing PID cases diagnosed up to 2019, divided by the average number of the Malaysian population over a specified time period.

## Results

A total of 4,838 articles were identified and screened in accordance with the pre-determined inclusion criteria. Thirty-four publications and 119 patients were included in this systematic review (Table 1). The study population was mainly Malays (67%), Chinese (14%), Indians (17%), Ibans (1%), and Melanau (1%). However, information regarding race was only available in 84 of 119 cases identified. The mean age was difficult to determine due to the nature of reporting by all the reports. Parental consanguinity was only recorded as present in three cases (2.5%). The pattern of major reporting centers, which were mainly from the public universities and the medical research team, was noted. Out of 63 cases identified with family history mentioned in the publications, only 38% had positive family history. Only two adult patients were reported to have PID (familial hemophagocycytic lymphohistiocytosis) (17), whereas the remaining aged between the 1st month of life and 17 years. However, patient age was only available in 26 publications.

**Table 1:** Primary Immunodeficiency Diseases Cases Reported in Malaysia.

**Paper ID**	**No of Patient/Gender**	**Age at onset of PID symptoms**	**Clinical presentation**	**Ethnicity**	**Infective agent**	**Clinical diagnosis**	**Treatment**	**Outcome**	**Genetic diagnosis/family history**
(8)	2 (Male)	Pt 1: 4 month-old	Hematemesis, purpura, eczema, bilateral otitis media	Chinese	*Salmonella enteritis, Pseudomonas septicaemia*, CMV	Pt 1: Wiskott Aldrich syndrome	–	Died	None. No family history
		Pt 2: 19 day-old	Staphylococcal sepsis, gastroenteritis, multiple episodes of epistaxis, hematemesis, purpura, rectal bleeding, eczema (at age 2.5 years)	Chinese	*Staphylococcal sepsis*, Hepatitis B	Pt 2: Wiskott Aldrich syndrome	Levamisole	Died	None. No family history
(9)	1 (Male)	20-month-old	Failure to thrive, multiple abscesses, frequent URTI, bilateral otitis media, scars from pyoderma on his thighs and the rest of his lower limbs	Indian	*Staphylococcus aureus, Pseudomonas aeruginosa*	Hyper IgM	Aggressive antibiotic therapy including cefuroxime, netilmicin and cotrimoxazole, and IVIG	Died	None
(10)	2 (Male)	Pt 1: 1 week-old	Frequent boils, recurrent urinary tract infection, septicemia, pneumonia, hepatosplenomegaly, cutaneous abscesses, anemic lymphadenopathy and failure to thrive	Malay	*Chromobacterium violaceum* and *Staphylococcus aureus*	Chronic Granulomatous Disease	Antibacterial therapy	–	None. Non-consanguineous families. Reported male siblings' death in early infancy, sparing female siblings
		Pt 2: 11 month-old	Jaundice, anemic, febrile, meningitis, hepatosplenomegaly, cutaneous abscesses, perianal abscesses, pneumonia and lymphadenopathy	Malay	*Staphylococcus aureus, Staphylococcus epidermis, E. coli*	Chronic Granulomatous Disease	Antibacterial therapy	Died	None
(11)	1 (Female)	12-month-old	Staphylococcal pneumonia left sided empyema. Had scaly skin and recurrent furuncles since aged 2 months old, cryptococcal meningitis	Chinese	*E. coli, Staphylococcus aureus, Cryptococcus neoformans*	Hyper-IgE syndrome	Prophylactic antibiotics (Fusidic acid, rifampin, ceftazidime, amikacin, intravenous amphotericin B, oral flucytosine	Died	–
(12)	2 (Male)	Pt 1: 2 years old	2 days history of fever and seizures, mildly jaundiced with generalized lymphadenopathy	–	Flavobacterium meningosepticum	Pt 1: X-linked lymphoproliferative disease (XLP)	Treated with chemotherapy for lymphoma	Died	None. Patients are siblings of non-consanguineous parents
		Pt 2: 5 years old	3 days history of fever, sore throat and generalized macular-papular rash, hepatomegaly, splenomegaly, lymphadenopathy	–	EBV	Pt 2: X-linked lymphoproliferative disease (XLP)	–	Died	
(13)	1 (Female)	40-day-old	Intermittent fever, hepatosplenomegaly, cytopenia, lymphadenopathy, mildly jaundice	Malay	–	Familial Hemophagocytic Lymphohistiocytoizis	–	Died	None. Consanguineous parents +
(14)	1 (Female)	3 month- old	Early childhood with recurrent infection of the sinopulmonary tract (recurrent otitis externa and chest infections; multiple cold staphylococcal abscesses of the skin; severe, extensive and pruritic eczematoid lesions of the skin and markedly elevated serum IgE). Coarse facies were not seen.	Chinese	*Staphylococcus aureus*	Hyper-IgE syndrome	Intravenous vancomycin, cotrimoxazole and fluconazole, IVIG. Intravenous ceftazidime, amikacin, amphotericin B and intensive chest physiotherapy (for lung)	Died	None. Consanguineous family + (her father's grandmother and mother's father are siblings)
(15)	2	Pt 1 and Pt 2	Bad impairment of other organ systems (renal failure and coagulopathy, severe pneumonitis, gastro-intestinal hemorrhage and hepatitis), bacteremia	–	Non-typhoidal Salmonella	Pt 1: Combined T and B cell deficiencies	–	Died	None
						Pt 2: Selective IgA deficiency			
(16)	3	Pt 1, 2 and 3	–	–	–	Wiskott–Aldrich syndrome	–	–	Pt 1(ID = P84): exon 2 missense 290C>T
									Pt 2(ID = P105a): nonsense exon 4, 400 C>A
									Pt 3 (ID = P168): exon 10 deletion 1115–1119 delc
(17)	2 brothers (Males)	Pt 1: 21 years old	Pyrexia of unknown origin, significant weight loss, hepatosplenomegaly	**–**	**–**	Familial hemophagocytic lymphohistiocytosis (FHL)	Intravenous cyclophosphamide, etoposide and prednisolone monthly for 6 months; subsequently maintained for oral cyclosporin for 1 year	Responded well to chemotherapy	None
		Pt 2: Twenties	One month history of fever, weight loss, febrile with herpes simplex infection on lips, multiple indurated plaque like lesion on lower limbs and trunk	**-**	**–**	Familial hemophagocytic lymphohistiocytosis (FHL)	Intravenous cyclophosphamide, etoposide and prednisolone monthly for 6 months; subsequently maintained for oral cyclosporin for 1 year	Responded well to chemotherapy	None
(18)	2	–	–	–	–	Familial hemophagocytic lymphohistiocytosis (FHL)	HSCT	–	–
(19)	1 (Male)	12 years old	Recurrent episodes of sepsis, multi-organ abscesses, infected non-healing ulcer and enlarged axillary lymph nodes	-	*Chromobacterium violaceum*	CGD	Oral antibiotics ciprofloxacin and cotrimoxazole	Alive	None
(20)	2 (1 Male, 1 Female)	–	–	–	Klebsiella pneumonia bacteremia	Pt 1: SCID	–	Died	None
		–	–	–	Candidiasis, Klebsiella pneumonia	Pt 2: SCID	–	Died	None
(21)	1 (Male)	2 years old	Recurrent infections with 2 episodes of chicken pox at age 2 years, repeated episodes of pneumonie and bronchiectasis by age 6 years.	Malay	Chicken pox	Partial defective BTK protein expression CD19 deficiency with selective IgM deficiency	IVIG	Alive	CD19 gene exon 1 A homozygous deletion of a C nucleotide (c.24delC)
			Physical examination revealed gross clubbing with crepitations at both lung bases						
(22)	2 (Pt 1: Male;	Born 1987	Meningococcemia, retinitis pigmentosa in the right eye, and a protracted episode that included herpes simplex, left foot cellulitis which grew Chromobacterium violaceum, hepatitis, a gastrointestinal hemorrhage, septicemia and oral candidiasis, discoid lupus erythematosus	Malay	*Chromobacterium violaceum*	Pt 1: p-47 phox deficient Chronic Granulomatous Disease	–	–	NCF1
	Pt 2: Female)	Born 1993	The affected younger brother-with Chronic Granulomatous Disease- (born 1993), succumbed to Chromobacterium violaceum septicemia complicated by intracranial bleeding just before this study was initiated.	Malay	*Chromobacterium violaceum*	Pt 2: Chronic Granulomatous Disease	–	Died	None
(23)	1 (Male)	27-day-old	Meningitis, BCG lymphadenitis, prolonged fever. Older brother had pneumonia at birth, recovered and well	Chinese	–	X-linked Chronic Granulomatous Disease	–	–	CYBB gene; Non-consanguineous family.
									Family study where older brother was both an X-linked Chronic Granulomatous Disease carrier and a Klinefelter.
(24)	52 patients (Cross-referencing to exclude overlapping cases)	-	The commonest clinical presentation is pneumonia (54%)	Malay (62%), Chinese (13.5%), Indian (23.1%), Iban (2%)	–	Predominant Antibody deficiency (40.4%) Phagocytic defect (17.3%), Combined immunodeficiencies (15.4%) and other cellular immunodeficiencies (11.5%).	–	–	Family history with a close family relative afflicted was a strong pointer to diagnosis for PID (52.6%)
(25)	1 (Male)	17 years old	Skin lesions, cellulitis, septicemia and microabscesses in the spleen and liver	-	*Chromobacterium violaceum*	Chronic Granulomatous Disease	Intravenous meropenem with cloxacillin, Emergency laparotomy	Died	–
(26)	1 (Male)	7 years old	Recurrent pyogenic infection, recurrent otitis media, recurrent bronchopneumonia, failure to thrive	Malay	–	X-linked Agammaglobulinemia with bronchiectasis	IVIG therapy, Inhaler prophylaxis	Alive	Base substitution in the first nucleotide of intron 9 and the mutation was IVS9+1G>C, Absence of BTK expression, circulating B cells and antibodies
(27)	1 (Male)	6-week-old	Chryseobacterium sepsis, hepatosplenomegaly, pancytopenia, increased histiocytes exhibiting hemophagocytosis, necrotizing enterocolitis	South Indian	*Chryseobacterium sepsis*	Griscelli syndrome	HLH-2004 protocol, HSCT	Alive	Homozygous mutation in RAB27A exon 6 c.550C>T Parents heterogenous carriers
(28)	2 (Male)	Pt 1: 18 month-old	Clinical features consistent with classic Wiskott Aldrich Syndrome for both patients (thrombocytopenia, eczema, and recurrent infections.	Malay	–	Pt 1: Wiskott–Aldrich syndrome	–	–	Novel mutation of c.28C>T in exon 1/Non-consaguineous parents, mother was carrier
		Pt 2: 4 month-old	Initial clinical presentations: Bleeding tendency. Thrombocytopenia and low mean platelet volume)	Malay	–	Pt 2: Wiskott–Aldrich syndrome	–	–	Nonsense mutation of c.264C>A in exon 2/ two older brothers died due to sepsis, mother was carrier
(29)	1 (Male)	8 month-old	Complex febrile fit, secondary to right otitis externa, chronic subacute arthritis of both knees	Not mentioned	*Pseudomonas aeruginosa*	X-linked Agammaglobulinemia	IVIG	Defaulted follow up after discharged from hospital	Base substitution g.36712A>G in the genomic DNA, *de-novo* splice site resulted in a truncated BTK protein
(30)	1 (Female)	4-month-old	Mutliple Intestinal Atresia that involved the pyloric, duodenal, jejunal, ileal and colonic segments. Antenatal ultrasound at 7 months of gestation showed polyhydramnios and Multiple Intestinal Atresia	Malay	–	TTC7A SCID	–	Died	Compound heterozygous mutations in *TTC7A*
(31)	1 (Male)	3-month-old	Hepatosplenomegaly, bronchopneumonia, multiple granulomas and abscesses in the lungs in the liver and spleen, fever, lymphadenopathy (18 months old)	Melanau	–	Autosomal recessive Chronic Granulomatous Disease	Multiple antibiotics, augmentin, doxycycline, co-trimoxazole and anti-TB (isoniazid, rifampicin and pyrazinamide)	HSCT was done 5 years after diagnosis	NCF1 (Neutrophil Cytolistic Factor 1). Eldest of two siblings of non-consanguineous parents. No family history of PIDs.
(32)	2	Pt 1: 1 years old	Monocyte with deficit of BTK expression	–	–	Pt 1: X-linked Agammaglobulinemia	IVIG therapy	–	BTK gene mutation, c.1888A>T
		Pt 2: 2 years old	Monocyte with deficit of BTK expression	–	–	Pt 2: X-linked Agammaglobulinemia	IVIG therapy	–	BTK gene mutation, g.34430_34447 delCAAAGTCATGATgtgagt
(33)	6	–	–	Malay	–	Pt 1: SCID	–	–	SCID
		–	–	Malay	–	Pt 2: SCID	–	–	SCID
		–	–	Malay	–	Pt 3: IL2RG SCID	–	–	IL2RG SCID
		–	–	Malay	–	Pt 4: SCID	–	–	SCID
		–	–	Malay	–	Pt 5: ADA SCID	–	–	ADA SCID
		–	–	Malay	–	Pt 6: IL2RG SCID	–	–	IL2RG SCID
(34)	1 (Female)	11 years old	High fever, recurrent episodes of diarrhea, oral thrush, and failure to thrive. PID screening showed low T cell, very low B cell counts, and low immunoglobulin levels	–	Cytomegalovirus	Combined T and B cell deficiencies with Cytomegalovirus retinitis	3 weekly intravenous immunoglobulins, cotrimoxazole prophylaxis, empirical therapy- syrup fluconazole and syrup nystatin, QID, also treated for Cytomegalovirus colitis, completed 6 weeks of intravenous ganciclovir	–	None
(35)	1 (Male)	10 month-old	Recurrent pneumonia, chronic diarrhea and failure to thrive	Malay	-	IL2RG SCID	Haploidentical HSCT	Alive	Heterozygous c.270-2A>T mutation in intron 2 of the IL2RG gene
									Non-consanguineous parents
(36)	1 (Female)	4-month-old	Recurrent abscesses over the whole part of the body, recurrent oral candidiasis, growth failure and recurrent pneumonias since 4 months old. She also had history of a few episodes of acute tonsillitis, chronic suppurative otitis media	Malay	Herpes zoster infection	Severe congenital neutropenia	Antimicrobial prophylaxis	Alive	Heterozygous variant in ELANE gene (c.640G>T; p.Gly214Ter
(37)	2	Pt 1 (Girl) and Pt 2 (Male)	Pt 1 and Pt 2 are siblings presented at different time points over a 20-year span with raised IgE levels (NIH score: 39), recurrent infections, eczema, hypereosinophilia and bronchiectasis	Malay	Cryptococcus	DOCK8 Immunodeficiency	–	–	Pt 1 and Pt 2: Large deletion in DOCK8 starting from exon 30 to 48 Consanguineous parents +
(38)	2 (Male)	–	–	–	–	Pt 1 and Pt 2: X-linked Agammaglobulinemia	IVIG replacement therapy	–	None
(39)	1 (Female)	12 years old	Recurrent fever, skin erythema and inflammatory arthritis	Malay	*Staphylococcus aureus*	Autoinflammatory disorder) NLRC4 mutation	–	Alive	Novel *de novo* heterozygous gain-of-function NLRC4 mutation (c.1970A>T, p.Gln657Leu)
(40)	1 (Male)	–	Family history of Chronic Granulomatous Disease	Malay	–	Chronic Granulomatous Disease	–	Alive	None
(41)	20 (3 patients were excluded due to duplicates with previous publications)	144 months	A cohort of patients with Inborn error of immunity who underwent HSCT in University Malaya Medical Centre from 1993 – 2018.	–	–	Chronic Mucocutaneous Candidiasis	HSCT	Alive	No genetic mutations described in this cohort.
		9 months		–	–	Kostmann	HSCT	Alive	
		12 months		–	–	SCID	HSCT	Alive	
		15 months		–	–	SCID	HSCT	Alive	
		144 months		–	–	Bruton	HSCT	Alive	
		56 months		–	–	Wiskott–Aldrich Syndrome	HSCT	Died	
		102 months		–	–	Cyclical Neutropenia	HSCT	Alive	
		24 months		–	–	Wiskott–Aldrich Syndrome	HSCT	Alive	
		9 months		–	–	Chediak Higashi	HSCT	Alive	
		12 months		–	–	SCID	HSCT	Alive	
		8 months		–	–	Wiskott–Aldrich Syndrome	HSCT	Alive	
		7 months		–	–	SCID	HSCT	Alive	
		12 months		–	–	SCID (Omenn)	HSCT	Alive	
		6 months		–	–	SCID	HSCT	Alive	
		48 months		–	–	X-linked Inhibitor of Apoptosis Protein defect (XIAP)	HSCT	Alive	
		20 months		–	–	SCID (Omenn)	HSCT	Alive	
		17 months		–	–	Familial Hemophagocytic Lymphohistiocytosis	HSCT	Alive	

*BCG, Bacille Calmette-Guerin; HSCT, Hematopoietic stem cell transplantation; IVIG, Intravenous Immunoglobulin; NIH, National Institutes of Health; Pt, Patient; SCID, Severe combined immunodeficiency diseases*.

**Figure 2:**
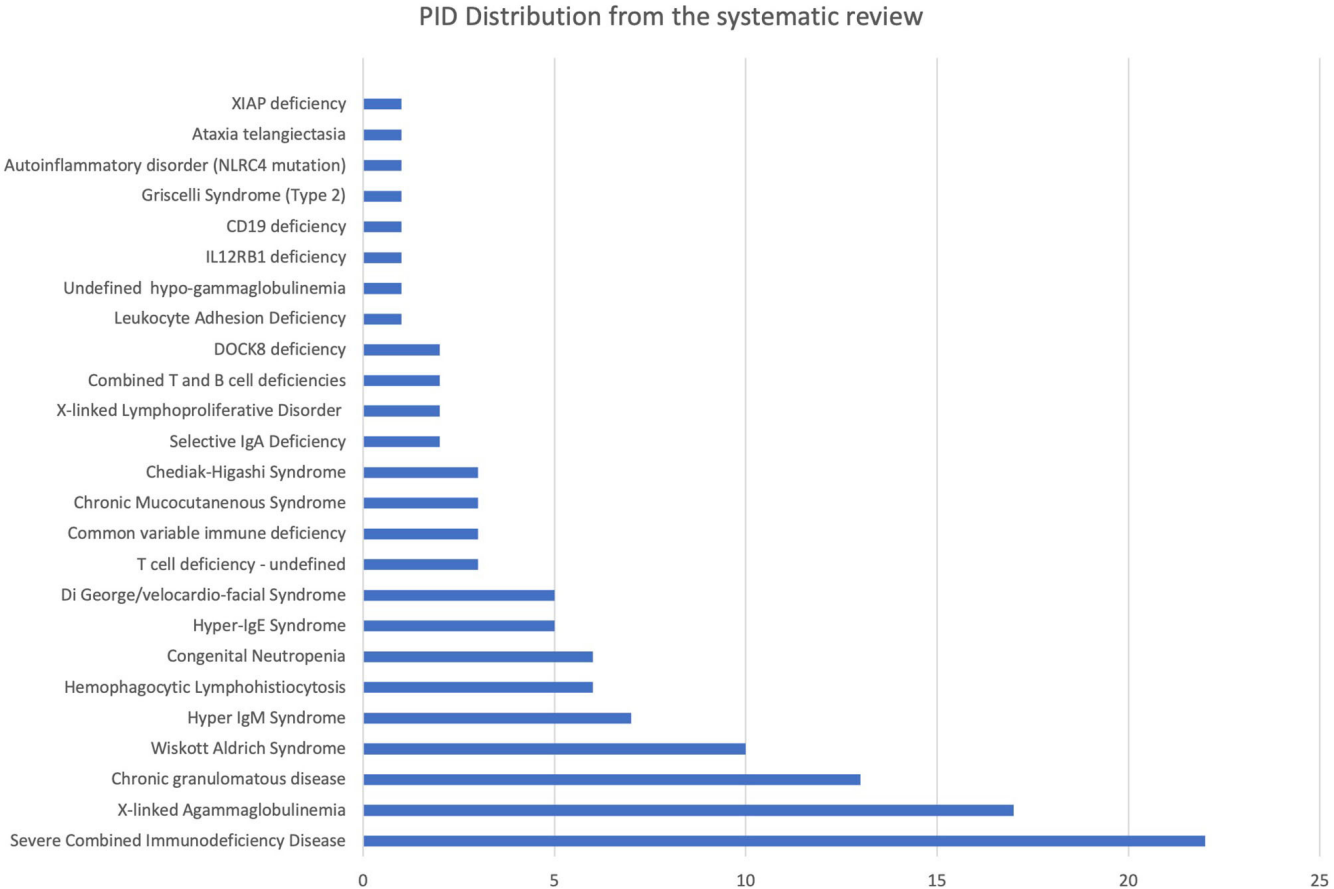
PID diagnosis distribution identified from systematic review.

A total of 119 PID cases were identified, and the diagnosis and distribution are illustrated in Figure 2. The most common forms of PID were immunodeficiencies affecting cellular and humoral immunity (30.3% of the cases published, Table 2), followed by predominantly antibody deficiencies (20.2%), congenital defects in phagocyte function and number (16.8%), diseases of immune dysregulation (10.9%), defects in intrinsic and innate immunity (3.4%), and autoinflammatory disorders (0.8%). The most common PID diagnosis was severe combined immunodeficiency (SCID, 22 patients), with genetic diagnosis available in 10 patients with SCID (Table 3). The second most common PID diagnosis was X-linked agammaglobulinemia (17 cases), followed by chronic granulomatous disease (CGD), affecting 14 patients (p47-phox-deficient CGD = three patients, X-linked CGD = two patients). The calculated prevalence rate was 0.37 cases per 100,000 population (from 1979 until 2019). We did not calculate the incidence as there was limited information in the literature.

**Table 2:** Spectrum of PIDs, 1979–2020.

**PID category**	***N***
**Immunodeficiencies affecting cellular and humoral immunity**	**36 (30.3%)**
Severe Combined Immunodeficiency Disease	22
Combined T and B cell deficiencies	2
Hyper IgM Syndrome	7
DOCK8 deficiency	2
T cell deficiency—undefined	3
**Combined immunodeficiencies with associated or syndromic features**	**21 (17.6%)**
Wiskott–Aldrich Syndrome	10
Ataxia telangiectasia	1
Di George/velocardio-facial Syndrome	5
Hyper-IgE Syndrome	5
**Predominant antibody deficiencies**	**24 (20.2%)**
X-linked Agammaglobulinemia	17
Common variable immune deficiency	3
Selective IgA deficiency	2
CD19 deficiency	1
Undefined hypo-gammaglobulinemia	1
**Disease of immune dysregulation**	**13 (10.9%)**
Hemophagocytic Lymphohistiocytosis	6
Chediak-Higashi Syndrome	3
Griscelli Syndrome (Type 2)	1
X-linked Lymphoproliferative Disorder	2
XIAP deficiency	1
**Congenital defects of phagocyte number or function**	**20 (16.8%)**
Congenital Neutropenia	6
Leukocyte Adhesion Deficiency	1
Chronic granulomatous disease	13
**Defects in intrinsic and innate immunity**	**4 (3.4%)**
IL12RB1 deficiency	1
Chronic Mucocutaneous Syndrome	3
**Autoinflammatory disorders**	**1 (0.8%)**
Autoinflammatory disorder (NLRC4 mutation)	1

**Table 3:** Genetic mutations described.

**Diseases**	**Number of patients**	**Mutations identified**
SCID	2	IL2RG
	1	IL2RG c.270-2A>T
	1	ADA
	1	PNP
	1	TTC7A
	1	ZAP70
DOCK8 deficiency	2	Large deletion in DOCK8 starting from exon 30- 48
WAS	1	WAS c.290C>T
	1	WAS c.400C>A
	1	WAS g. 1115–1119 delC
	1	WAS c.28C>T
	1	WAS c.264C>A
XLA	1	BTK g. IVS9+1G>C
	1	BTK g. 36712A>G
	1	BTK c.1888A>T
	1	BTK g.34430_34447 delCAAAGTCATGATgtgagt
CD19 Deficiency	1	CD19 c.24delC
Griscelli Syndrome (Type 2)	1	RAB27A c.550C>T
CGD	2	NCF1
	1	CYBB
Congenital neutropenia	1	ELANE c.640G>T
Autoinflammatory syndrome	1	NLRC4 c.1970A>T

Genetic tests are not universally available in Malaysia at present, and most are paid for by patients and families. Genetic mutation results were identified in 26 patients (21.8%; Table 3). Twenty-two patients underwent hematopoietic stem cell transplantation in Malaysia, whilst 19 received intravenous immunoglobulin replacement therapy. Only 28 patients were documented as alive, and 16 had died at the time of publication of the case reports. However, no data were available for the remaining publications.

In order to place results from Malaysia in context, a similar comparison of PID diseases frequencies was made, using published reports from other countries (Table 4). The prevalence of PID in Malaysia using this method is the lowest compared to reports from other nations. This low prevalence comes from a systematic review in the absence of national registry or cohort report, as reported by other countries.

**Table 4:** The comparison of primary immunodeficiencies prevalence, frequencies, consanguinity, and availability of genetic results with various countries.

**Country**	**Malaysia**	**Singapore (42)**	**Korea (43)**	**Taiwan (44)**	French (45)^**^	**Brazil (46)**	**Kuwait (5)**	**USA (47)**
Time of reporting	1979–2020	1990–2000	2001–2005	1985–2004	2005–2009	1978–2011	2004–2018	1992–2020
Types	Systematic review	Cohort report	National registry	National registry	National registry	Cohort report	National registry	National registry
Number of cases	119	39	152	37	3,083	1,008	314	5,484
Incidence	–	2.65 per 100,000 live births	–	2.17 per 100,000 livebirths	–	–	24.96 per 100,000	–
Prevalence	0.37 cases per 100,000 population	–	11.25 per million children	1 in 46,000 live births	4.4 per 100,000 inhabitants	–	^*^20.27 per 100,000 population	–
Consanguinity	2.5%	Not reported	Not reported	0%	15%	Not reported	78%	Not reported
Genetic mutations known	21.7%	Not reported	Not reported	16%	40%	Not reported	69%	46%
Immunodeficiencies affecting cellular and humoral immunity	30.3%	10.3%	13.2%	11%	17.2%	6.7%	31.8%	9.6%
			^*^T cell deficiencies 4.6%	^*^T cell deficiencies 19%				
Combined immunodeficiencies with associated syndromic features	17.6%	12.8%	–	–	14%	8.3%	21.7%	20%
Predominantly antibody deficiencies	20.2%	41%	53.3%	46%	42.8%	60.8%	17.8%	49.2%
Diseases of immune dysregulation	10.9%	–	–	–	6.6%	5.3%	15%	4.2%
Congenital defects of phagocyte number or function	16.8%	15.4%	28.9%	24%	18.6%	8.7%	6.4%	12%
Complement deficiencies	–	–	–	–	0.5%	2.9%	7%	0.5%
Autoinflammatory Syndromes	0.8%	–	–	–	–	1.3%	0.3%	–
Defects of innate immunity	3.4%	–	–	–	0.2%	5.9%	–	–
Others	–	17.9%	–	–	–	–	–	2.2%

* cumulative incidence.

** *percentage was calculated from results presented in the study (Table 2) (45)*.

## Discussion

To the best of the authors' knowledge, this review is the first to study the prevalence of PIDs in Malaysia, which was 0.37 per 100,000 population. These rates were comparably lower than published rates from other countries, where the prevalence rate was ~4.4–20.27 per 100,000 population (5, 42–47).

In addition, these rates were lower than those in several neighboring countries in Southeast Asia with similar geo-political environments. Singapore has an incidence rate of 2.65 per 100,000 live births and an estimated occurrence rate of one in 37,000 live births (47). Thailand reported a cohort of 67 patients with PID from 18 year-experience from a single tertiary centre (48). No national PID patient registry databases are available in Southeast Asian countries. The only countries in the Asia Pacific with national PID registries are Japan, South Korea, Taiwan, Hong Kong, and China (49). The Middle East countries with national PID registries are Kuwait, Turkey, and Iran (5, 50, 51).

There was an increase in reporting over time which may be attributed to the increase in numbers of practicing clinical immunologists, enhanced diagnostic strategies, and facilities and improved awareness amongst medical fraternities locally (52).

This systematic review found that parental consanguinity was 2.5%, which was comparable with the parental consanguinity rate of 2.4% in a Malaysian population study on birth defects (53). Consanguineous marriage is not widely practiced in Malaysia compared with other South Asian and Middle East countries but is practiced in certain ethnic groups or indigenous populations.

This study is unique because it was not based on a national registry. However, it was the best attempt in delineating the prevalence rates of PIDs in Malaysia. Systematic review offers a non-biased approach to reviewing the evidence available in the absence of a national patient registry database. Furthermore, this systematic review is a novel approach in assessing PID prevalence in a low resource country that does not have a national database and a model that could be followed by other countries, such as in South America and Africa.

The potential limitations of this study were the calculations that were based on published evidence. The prevalence rate may not reflect the actual rate, because many PID cases are undiagnosed and, even if diagnosed correctly, not published. Furthermore, reporting bias possibly existed, where certain diagnoses were reported more than others. This bias could explain why the most common PID diagnoses in this systematic review were severe combined immunodeficiencies rather than primary antibody defects, which are more prevalent in patient registry databases worldwide. Furthermore, only two adult patients were reported, and only three Common Variable Immunodeficiency cases were published, thereby emphasizing that the lack of clinical immunologists to take care of adult patients may be a major contributing factor for low adult PID cases diagnosed or not reported.

Therefore, a national PID registry database must be urgently established in Malaysia. Inter-collaboration between various agencies is the manner to move forward in terms of improving access to early diagnosis and treatment of PIDs in Malaysia.

## Conclusions

The prevalence of PID is lower in Malaysia than in other geographical regions. The comparison of PID prevalence between countries may be misleading due to various case-finding strategies. Efforts should be geared toward setting up a national PID patient registry database in Malaysia.

## Data Availability Statement

The datasets presented in this study can be found in online repositories. The names of the repository/repositories and accession number(s) can be found in the article/Supplementary Material.

## Author Contributions

NA, IH, and ZZ performed the literature search, reviewed the title, and abstracts independently and conducted the stratifications. IA and NA conducted the data extraction of relevant studies. Any disagreements/clarifications were confirmed with AG. The prevalence rates were calculated by EM. IA wrote the first draft of the manuscript. All authors designed the study. All authors critically reviewed the literature search and revised the manuscript. All authors read and approved the final version of the manuscript.

## Conflict of Interest

The authors declare that the research was conducted in the absence of any commercial or financial relationships that could be construed as a potential conflict of interest.
