# Supplementary material for: Systematic Review of Primary Immunodeficiency Diseases in Malaysia: 1979–2020
Source: Front Immunol. 2020 Aug 26;11:1923. doi: 10.3389/fimmu.2020.01923 (PMC7479198; doi:10.3389/fimmu.2020.01923)
Supplement: Supplementary file 1 [file Data_Sheet_1.PDF]

Supplement 1

Main reasons of 4737 abstracts excluded from the systematic review analysis:

1. Secondary immunodeficiencies = 944 abstracts
2. Animal subjects = 1690 abstracts
3. Plant subjects = 226 abstracts
4. Knowledge, attitude, practice (KAP) study = 387 abstracts
5. Physics term (ie: protons, ions, photon, nanoparticles, radiation) = 621 abstracts
6. Technology = 260 abstracts
7. Environment (Soil, water, air pollution) = 609 abstracts
