# Supplementary material for: Systematic Review of Primary Immunodeficiency Diseases in Malaysia: 1979–2020
Source: Front Immunol. 2020 Aug 26;11:1923. doi: 10.3389/fimmu.2020.01923 (PMC7479198; doi:10.3389/fimmu.2020.01923)
Supplement: Supplementary file 2 [file Data_Sheet_2.PDF]

Supplement 2

PubMed search algorithms:

Search: ((“primary immunodeficiencies disease*” OR “immunologic deficiency syndrome*” OR “chronic granulomatous disease” OR CGD OR “severe combined immunodeficiency disease” OR SCID OR “hemophagocytic lymphohistiocytosis” OR “immune dysregulation” OR “IVIG replacement therapy” OR “intravenous immunoglobin replacement therapy” OR bronchiectasis OR “antibody deficiency” OR “combined variable immunodeficiency” OR CVID OR “inflammatory bowel disease” OR IBD OR “hyper IgE Syndrome” OR
“mendelian susceptibility mycobacterium” OR “bruton tyrosine kinase” OR BTK OR “tyrosine kinase 2 deficiency” OR TYK2 OR STAT* OR IL* OR interferon* OR cytokine* OR “lymphocyte subsets” OR TBNK OR “auto-inflammatory” OR “auto-immunity” OR “hyper IgM Syndrome” OR “T cell defect” OR “B cell defect” OR “chronic mucocutaneous candidiasis” OR “immune deficiency”) AND Malaysia)
